# A CBCT Based Three-Dimensional Assessment of Mandibular Posterior Region for Evaluating the Possibility of Bypassing the Inferior Alveolar Nerve While Placing Dental Implants

**DOI:** 10.3390/diagnostics10060406

**Published:** 2020-06-14

**Authors:** Mohammed G. Sghaireen, Kumar Chandan Srivastava, Deepti Shrivastava, Kiran Kumar Ganji, Santosh R. Patil, Anas Abuonq, Mohammed Assayed Mousa, Najla Dar-Odeh, Ghazi M. Sghaireen, Mohammad Amjad Kamal, Mohammad Khursheed Alam

**Affiliations:** 1 Prosthodontics, Prosthetic Dental Sciences, College of Dentistry, Jouf University, Sakakah 72345, Saudi Arabia; dr.mohammed.sghaireen@jodent.org (M.G.S.); dr.mohammed.mousa@jodent.org (M.A.M.); 2 Oral Medicine & Radiology, Department of Oral & Maxillofacial Surgery & Diagnostic Sciences, College of Dentistry, Jouf University, Sakakah 72345, Saudi Arabia; drkcs.omr@gmail.com (K.C.S.); dr.santosh.patil@jodent.org (S.R.P.); 3 Periodontics, Department of Preventive Dentistry, College of Dentistry, Jouf University, Sakakah 72345, Saudi Arabia; sdeepti20@gmail.com (D.S.); dr.kiran.ganji@jodent.org (K.K.G.); 4 Department of Prosthodontics, King Fahad Military Medical Complex, Dhahran 31932, Saudi Arabia; anas.abouonq@gmail.com; 5 Department of Oral Basic and Clinical Sciences, College of Dentistry, Taibah University, Al Madinah Al Munawarah 42353, Saudi Arabia; najla_dar_odeh@yahoo.com; 6 Department of Oral Surgery, Oral Medicine and Periodontics, School of Dentistry, University of Jordan, Amman 11942, Jordan; 7 Department of General Medicine, Al Rajhi Medical college, Al Bukayriyah 52736, Saudi Arabia; ghazimohad17@gmail.com; 8 King Fahd Medical Research Center, King Abdulaziz University, Jeddah 21589, Saudi Arabia; 9 Enzymoics, 7 Peterlee Place, Hebersham, NSW 2770, Australia; 10 Novel Global Community Educational Foundation, Hebersham 2770, Australia; 11 Orthodontic Division, Preventive Dentistry Department, College of Dentistry, Jouf University, Skaka 72345, Saudi Arabia; dr.mohammad.alam@jodent.org

**Keywords:** conebeam computed tomography, dental implant, buccolingual, inferior alveolar nerve canal tracing, inferior alveolar nerve injury

## Abstract

A high rate of nerve injury and related consequences are seen during implant placement in the posterior mandibular arch. An approach has been proposed to avoid nerve injury by dodging the inferior alveolar nerve (IAN) while placing an implant. A prospective study with a total of 240 CBCT (cone beam computed tomography) images of patients with three dentate statuses, namely, edentulous (group I), partially edentulous (group II) and dentate (group III) were included in the study. The nerve path tracing was done on CBCT images with On-demand 3D software. The three dimensions, i.e., the linear distance from the outer buccal cortical plate to the inferior alveolar nerve (BCPN), linear distance from the outer lingual cortical plate to the inferior alveolar nerve (LCPN) and linear distance from the midpoint of the alveolar crest to the inferior alveolar nerve (ACN) were assessed. The data were presented and analyzed between variables using one-way ANOVA and independent *t*-test in SPSS version 21.LCPN of the right 1st premolar region (*p* < 0.05) was significantly different among the groups with edentulous subjects recorded with the minimum value (6.50 ± 1.20 mm). Females were found to have significantly (*p* < 0.05) less available bone (6.03 ± 1.46 mm) on the right side of the mandibular jaw compared to males in edentulous group of patients. On comparing age groups for partially edentulous subjects, LCPN of the right 1st premolar region had significantly (*p* < 0.05) less available bone (6.03 ± 0.38 mm) in subjects with age ≥54 years. The IAN follows a lingual course in the molar region and later flips to the buccal side in the premolar region. The LCPN dimension in the 1st and 2nd premolar region was found to be more than 6 mm irrespective of age, gender and side of the jaw. Thus, it can be considered as a suitable site for placing implants while bypassing the IAN with CBCT assessment remaining as the mainstay in the pre-surgical phase.

## 1. Introduction

Pre-operative surgical planning is a pre-requisite for successful dental implant (DI) therapy. The selection of an appropriate imaging modality along with the expert’s judgment about the clinical scenario aids in the treatment planning. The functional aesthetics at the site of missing dentition ought to be contemplated in terms of patient aesthetic desire, anatomical variations and patient’s systemic status. Various radiographic techniques give an insight intovital anatomical structures especially in the mandibular arch which play a crucial role in any surgical procedures with regards to osteotomy, nerve repositioning andridge split for implant placement [1]. It is imperative for a clinician to study bone density, thickness and integrity of the cortical bone and also the optimal position and orientation of the DI in addition to the vital anatomic structures.

Three-dimensional (3D) digital radiography has been used predictably and with high success rates in clinical practice [2,3]. The advent of cone beam computed tomography (CBCT) imaging has expanded the doors for analyzing morphologic parts of the head and neck structures, including alveolar bones and nerve canals [1]; however, confinements of the innovation have yet to be characterized. CBCT caters for the oral and maxillofacial region in different anatomical planes, thus its application can precisely estimate the stature and thickness of the alveolar bone where an implant is intended to be placed [4]. Selecting the osteotomy site for the placement of DI can sometimes be challenging to the clinician because of the close proximity to the inferior alveolar nerve (IAN), mental foramen (MF) and maxillary sinuses.

Placement of the DI in the partially dentulous as well as edentulous mandibular arch can sometime be a nightmare for the clinician because of the variation in the course of the IAN and its exit from the MF. To address the above-mentioned issue, few cadaveric [5,6,7], as well as human studies [8,9,10] have been done.

The course of the inferior alveolar nerve (IAN) and its relation to the teeth has been studied by several investigators [10,11,12]. The variation in the location of MF and pattern of inferior alveolar nerve canal (IANC) has been studied by various investigators in different populations [13,14,15,16,17].

Despite various studies on this issue, the following limitations can be outlined. Firstly, most of the studies have made the coronal measurement from IAN up to the root apex [8,10,18,19] knowing the fact that the location of the apex will vary due to the inherent root dilacerations. Moreover, in the clinical scenario of DI placement, vertical measurement from the crestal bone as reference point is considered. Secondly, previous studies have excluded the dimensional appraisal of IAN in the third molar area, which being almost the first region where IAN appears in the arch and bears a clinical relevance. The surveys made in the earlier studies were limited to the dentate patients and very few studies included partially and completely edentulous patients with the sole aim of tracing the course of the IAN [20]. Furthermore, studies did not appear to be in agreement on the dimensional variation on either side of the arch between the sample characteristics like gender and age [10,19,20,21]. This information, if made available will aid the clinician to successfully place the DI in the region with sufficient dimension of bone without running the risk of injury to the IAN.

It has been found that due to the resorption of the alveolar bone in bucco-lingual and apico-coronal directions, it is difficult to place a longer implant of wider diameter in such areas. Moreover, in severely resorbed ridges the apico-coronal height compromises the placement of the DI parallel to the long axis. In addition to this, a close proximity to the vital structures makes it even more difficult for the placement of the DI. To avoid any extensive surgical procedures such as nerve repositioning or ridge augmentation in the above-mentioned scenario, a modification in the path of implant placement in either of the directions can be justified. If a small diameter implant is planned to be placed in either buccal or lingual directions with the help of a surgical guide provided sufficient bone (6 mm) is available, it can bypass the nerve without injuring it [22].

The gravity of this projected technique of DI placement prompted us to conduct the present study. The aim of the study was to assess the availability of sufficient bone (≥6 mm) for the placement of DIs via measuring the distance between the IAN to their corresponding outer cortical plates in either direction as well as the alveolar bone height above the nerve throughout its course, from the third molar to the MF.

The null hypothesis which needs to be tested with the current study states that there is an equal amount of bucco-lingual bone presentin the osterior region of the mandibular arch in all types of dentate state of patients to place a dental implant.

## 2. Materials and Methods

### 2.1. Study and Sample Characteristics

**Figure 1:**
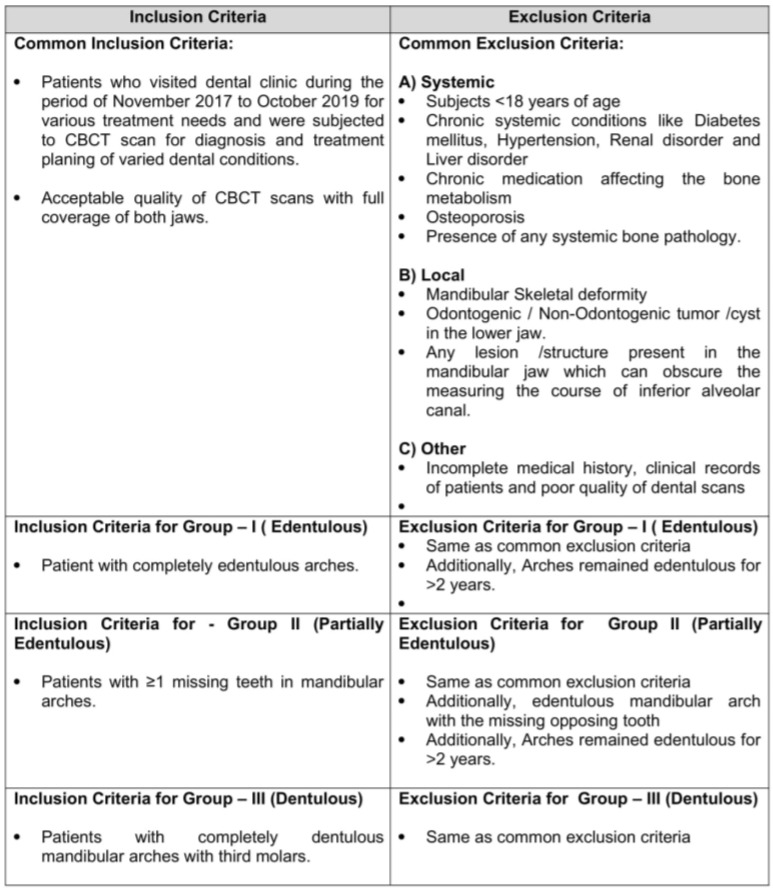
Inclusion and exclusion criteria.

An observational study in a hospital-based setting was conducted. Ethical approval was procured by the local bioethics committee (08-09/41). This prospective study involved image analysis of CBCT scans taken for the patients who visited the dental clinic from November 2017 to October 2019 for various treatment needs. The scans were taken in accordance with the standard protocol for the purpose of diagnosis and treatment planning of varied dental conditions. Based on the dentition status of patients, groups identified in the study were edentulous (group I), partially edentulous (II) and dentulous (III), with each group comprising of 80 samples (Figure 1).The current study involving human participants was reviewed and approved by the ethical board. Written informed consent for participation was taken for this study in accordance with the national legislation and the institutional requirements. The data collected from this research is solely for research and educational purposes.

**Figure 2:**
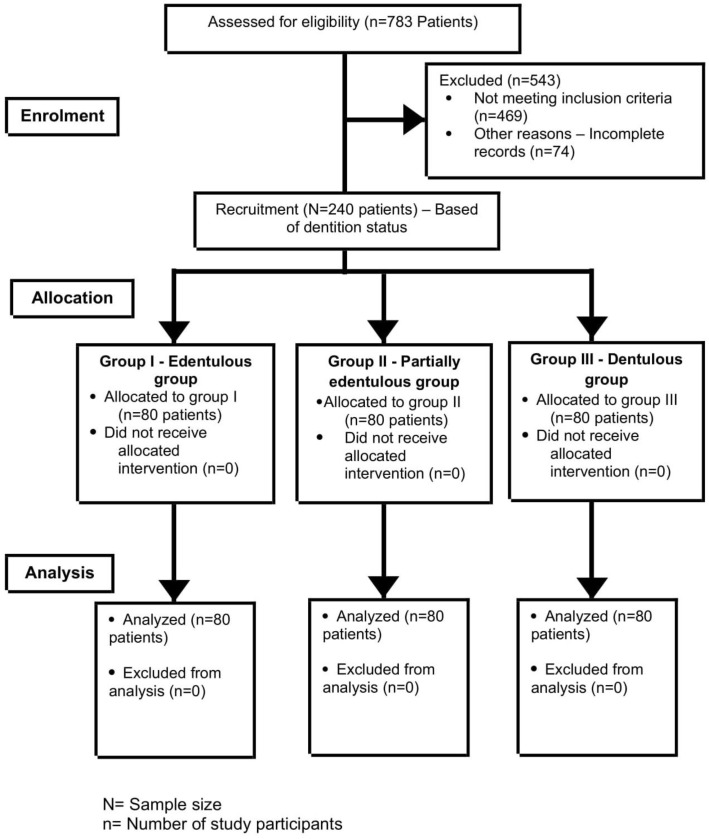
Showing STROBE (Strengthening the Reporting of Observational Studies in Epidemiology) statement.

Collectively, a total of 240 scans were considered eligible based on the inclusion and exclusion criteria of the study (Figure 2). The current study was conducted in accordance with the Strengthening the Reporting of Observational Studies in Epidemiology (STROBE) guidelines [23].

### 2.2. Specifications of CBCT Machine and Workstation

This radio graphicstudy investigated the course of IAN beginning at the third molar till its termination at MF on the CBCT scans. The scans were taken with SCANORA 3Dx (Nahkelantie 160, Tuusula, Finland) with standard operating specifications (90 KV and 10 mA). For including both jaws in the scan, medium field of view (FOV = 80 × 100) with standard resolution mode (voxel size of 0.25 mm) was selected. The protocol of image acquisition involved informing the patient about the procedure in detail, followed by which a protective shield was worn by the patient. The complete scan time involved was 20 s which consisted of 360° rotation of X-ray–receptor assembly around the static patient. For viewing and interpretation of images, the workstation was equipped with On Demand 3D software version 1.0.10.6388 (Yuseong-gu, Daejeon, Korea). The images are displayed on aTFT 27-inch monitor with 1280 × 1024-pixel screen resolution.

### 2.3. Standardization of Examiners and Reliability Analysis

Image analysis was carried out by two independent oral and maxillofacial radiologists. To ensure consistency in the interpretation, the examinations were initially done by open discussion to standardize their measurement methods. Later, statistical tools were employed to assess the test–retest reliability with 10 samples (later not included in the study). The inter- and intra-examiner reliability was found to be 0.86 and 0.91, respectively, which shows a strong level of agreement [24].

### 2.4. Study Protocol and Image Analysis

Based on the inclusion and exclusion criteria, prospective patients in all age groups were selected for the study. They were informed about the objectives of the study in understandable language and written informed consent was obtained later. Their participation in the study was voluntary and they were given the option to leave the study at any given point in time. The biographic and demographic data including age, gender and nationality were collected along with the medical and dental histories. Complete oral examination was done to assess the dentition and periodontal status. Finally, the CBCT scans, with the standard protocols, were taken for all the participants.

**Figure 3:**
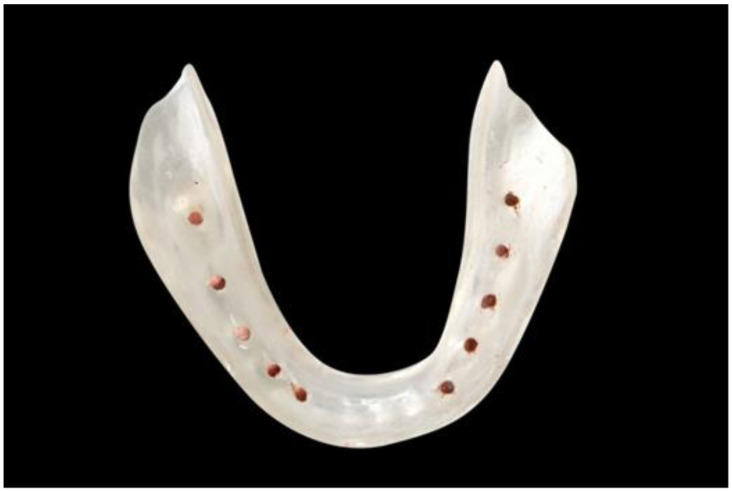
Diagnostic stent made of self cure acrylic with channels filled with gutta-percha.

**Figure 4:**
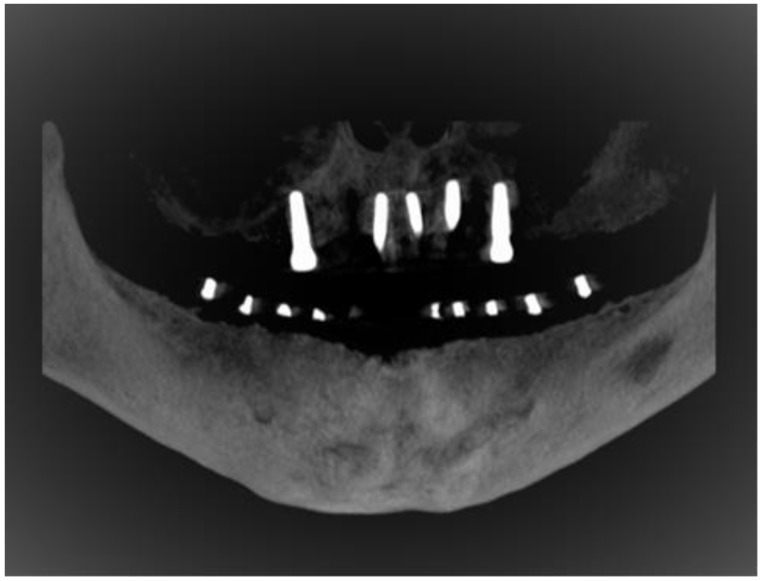
Reformatted panoramic view on CBCT for edentulous mandibular ridge with customized stent.

For the accurate identification of the tooth region in completely edentulous patients, a base plate made of clear acrylic was made. Later, the gutta-percha sticks were incorporated through the tissue bearing surface at the base of the acrylic plate and then CBCT scans were taken (Figure 3 and Figure 4). For other groups, scans were taken in the usual manner.

**Figure 5:**
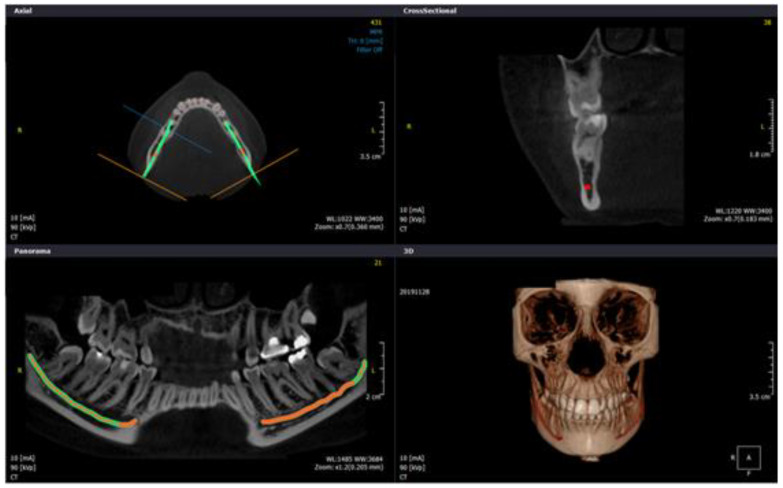
Images of IAN tracing shown using On-Demand 3D computer software with axial plane, panoramic view, transverse cuts and 3D view for dentate subject.

**Figure 6:**
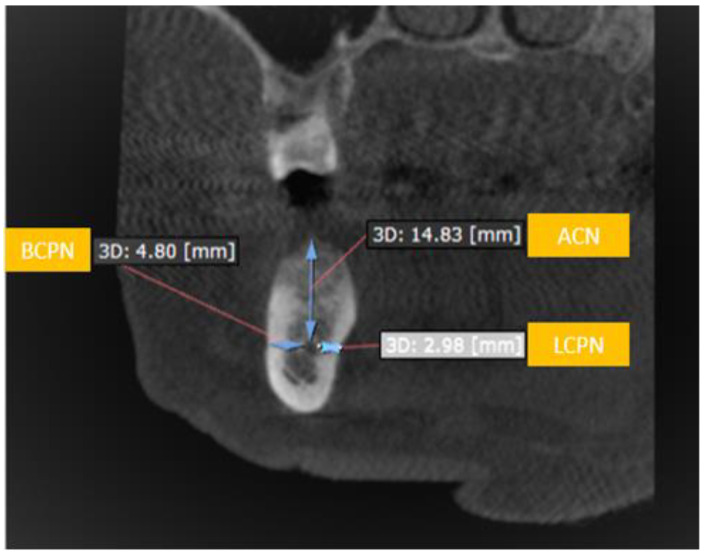
Showing the distance from outer buccal cortical plate to the inferior alveolar nerve (BCPN), outer lingual cortical plate to inferior alveolar nerve (LCPN) and alveolar crest to inferior alveolar nerve (ACN).

The IANs were traced on CBCT scan imageswith the assistance of computer software (On Demand 3D, Yuseong-gu, Daejeon, Korea). Panoramic reconstruction of the views was created in 3 mm thickness and 0.5 mm cross-sectional cuts for the area under investigation. Tracing and measurements were taken from the third molar till the nerve leaves through the MF (Figure 5). The following three measurements were recorded in respect to the IAN sited at each posterior tooth region for both sides of the jaw (Figure 6).

I. Buccalcortical plate to IAN dimension (BCPN): Linear distance from the IAN to the nearest corresponding buccal outer cortical plate.
II. Lingual cortical plate to IAN dimension (LCPN): Linear distance from the IAN to the nearest corresponding lingual outer cortical plate.
III. Alveolar crest to IAN dimension (ACN): Linear distance from the IAN coronally to the midpoint of alveolar crest bone corresponding to the long axis of tooth.

Measurements were made at the level of the bisecting line of all teeth from third molar until the MF. If any tooth was extracted in study group II, the measurement was made at the level of the line bisecting the opposing tooth. 

### 2.5. Statistical Analysis

#### 2.5.1. Sample Size and Power of the Study

With regard to the sample size, post hoc analysis was carried out using software G Power 3.1.9.2 (Heinrich Heine Universität, Düsseldorf, Germany). ANOVA: fixed effects, one way was chosen as the statistical test in the F-tests family. The post-hoc test was computed with a confidence interval of (α) 0.05, effect size of 0.25 and for 3 study groups. The sample size achieved a statistical power of 0.94.

#### 2.5.2. Data Entry, Descriptive and Inferential Analysis

Data editing and coding was done using a Microsoft-excel spreadsheet. Baseline characteristics of sample were depicted in number and percentages, whereas the spatial measurements of IAN course along the arch were represented in mean and standard deviation (SD). Testing of hypothesis was performed using one-way ANOVA (analysis of variance) and independent *t*-test at a 95% confidence interval (CI). Association was considered statistically significant when *p* < 0.05. Statistical package for social sciences (SPSS) version 21 was used for all statistical analysis.

## 3. Results

### 3.1. Descriptive Statistical Analysis of the Baseline Sample Characteristics

The current study had three study groups with each group having a sample of 80 subjects, thus accounting for a total sample size of 240. Dentulous and partially edentulous groups were further subdivided into the specified age slabs. Nearly equal representation of age categories and gender was made in the study groups (Table 1).

### 3.2. Inferential Analysis of the BPCN, LCPN and ACN Among the Study Groups

In the present study, we undertook three dimensions (BCPN, LCPN and ACN) at intervals corresponding to each posterior tooth. They were analyzed to aid in assessing the course of the IAN in the mandibular arch. These region-specific dimensions were compared to their corresponding values among the study groups and sides of the mandibular jaw. Within the group, a trend of decreasing BCPN with simultaneous increase in LCPN dimension was found as the IAN takes its course from 3rd molar towards 1st premolar. Although statistically non-significant (*p* > 0.05), this pattern was seen across all the groups. Exceptions were reported for LCPN dimensions in the right 1st premolar region (*p* < 0.05) and BCPN dimensions in the right 2nd premolar region (*p* < 0.01) (Table 2). Regarding ACN dimension, statistically significant (*p* < 0.001) variation was seen among the study groups with edentulous having the lowest value.

### 3.3. Inferential Analysis of the BPCN, LCPN and ACN among the Various Independent Variables-Side of Jaw, Gender and Age

Comparisons of BCPN, LCPN and ACN dimensions between the genders yielded statistically significant mixed variation at different significance levels (*p* < 0.05/< 0.01/< 0.001). On further exploration, it was found that in the majority of occasions, males had significantly more bone compared to females (Table 3).

Comparisons of dimensions were also done between the different age categories of the study group consisting of partially edentulous and dentulous patients. At varying significance levels (*p* < 0.05, *p* < 0.01, *p* < 0.001), the dimensions showed variations among age groups, with the younger age group (18–35 years) outscoring the other age groups in the majority of events. Edentulous showed significantly less available bone among groups (Table 4).

Despite, significant variation reported at different levels with age, gender and side of the jaw, the mean dimension of LCPN at 1st and 2nd premolar regions was consistently reported to be above 6 mm.

## 4. Discussion

During implant placement, cognizance of vital structures and anatomical landmarks are of considerable importance. Implant osteotomy in the posterior region of the mandible is even more crucial because of the extended course of the IAN within the neurovascular bundle. Literature has supported the fact that in mandibles, implant related nerve injury to IAN ranges from 0–40% [22], where as dysesthesia after implant placement was reported in 1.7–43.5% of cases and 5–15% of cases reported having permanent sensory disturbance [25].Thus, as a clinician, pre-operative assessment of the nerve course in mandibles can overcome the complications associated with impingement or compression of the IAN.

Various imaging modalities are available to facilitate this section of the pre-operative phase of implant surgery. Previously, the most popularly used modality for this purpose was panoramic imaging. The major drawback which limits its use is mainly two fold [1]. It depicts three dimensional dental arches as two-dimensional structures, thus missing its bucco-lingual dimension. Superimposition of anatomical structures also adds to the difficulty in pre-operative assessment of the arches. The initial revolution in imaging came with the advent of computed tomography (CT) [26]. Despite being a sectional imaging, limited accessibility to the dental patients and high radiation dose, has confined its use in implant imaging [1,4,27]. CBCT brought a drastic revolution in the dental implant imaging. It gives volumetric data of curved arches in a single rotation by exposing the patient to considerably reduced radiation within the vicinity of dental setup. The images obtained from CBCT can be reformatted to all three planes; sagittal, axial and coronal along with reformatting volume rendered images. With the help of interactive software available with the CBCT, it makes it possible to extract clinically applicable information, like measurements, nerve tracing and even facilitates performing mock implant surgery [2,4,28,29,30]. Considering the above-mentioned merits of CBCT, it was selected as the tool for image analysis in the present study.

In order to avoid complications related to nerve injury, either pre-surgical planning or surgical intervention such as nerve repositioning can be done. Pre-procedural planning with the help of CBCT, ascertains the position of the IAN and the amount of available bone, thus helps in selecting a suitable implant site [2,9,30,31]. The clinical scenario becomes challenging, when the vertical quantity of bone is not sufficient. This situation arises commonly when we are dealing with partially or completely edentulous arches. These arches have undergone a considerable amount of resorption and thus leave behind a small amount of bone above the IAN. To deal with such situations, the authors of the present study intend to propose a novel clinical approach to detour dental implant placement without injuring the IAN.

### 4.1. Comparison of BCPN, LCPN and ACN Dimensions among the Patients with Different Dentition Status

Three dimensions namely BCPN, LCPN and ACN were measured and analyzed to serve the objectives of the present study. The dimensional computations were initially utilized to study and explore the course of the IAN starting from the mandibular third molar up till the first premolar of the same side of the arch. Further appraisal of these measurements was done to explore the feasibility of placing an implant in either the buccal or lingual aspect of the IAN. This assessment was positioned on the fact that there should be enough sound bone available in the area to accommodate the small diameter implant which is approximately 3 mm. Consideration is also given to the concept which states that there should be ahealthy bone of about 1.5 mm from eachimplant to the IAN and nearest cortical plate respectively [32,33,34]. Comprehensively, it can be speculated that if aminimum 6 mm of bone is available buccally or lingually to the IAN; dental implants can be placed without running the risk of injuring the IAN (Figure 5). Although conflicting results are available in literature [18,35].

Studies pertaining to these dimensions in relation to the course of the IAN have primarily been done with dentate or partially edentulous subjects. Hence, there is a lacuna in the literature about similar data for edentulous subjects. The present study thus attempts to bridge the gap in the literature, by including three classical types of dentition status.

The measurements were taken from IAN sited at the mid-point of the long axis of each posterior tooth to its respective buccal (BCPN) and lingual (LCPN) cortical plate. The dimension of the LCPN was reported to be at its least at the 3rd and 2nd molar region, which drastically starts increasing from the first molar region onwards. The maximum chunk of the LCPN was reported in the 2nd and 1st premolar region. The converse situation was seen with respect to the BCPN. It was measured at its maximum at the 3rd molar and minimum at the premolar region. At times, it has been seen that at the 1st premolar there was no BCPN measured from IAN to the corresponding cortical plate. In such cases, it was understood that either the IAN had made an exit from the MF [19,28,29] situated between the premolars or at the apex of 1st premolar. However, the commonest position of the MF in the Saudi population was found to be in line with the long axis of the second premolar [13,14]. It has been seen that the commonest pattern for IANC is linear in nature and represent 46.2% of the cases, whereas in 15.2% of the cases it was found to have an anterior loop pattern [13]. The inference drawn from the pattern of BCPN and LCPN is that the IAN has taken the lingual course to the molars and haslater adopted a buccal direction. This observation is in agreement with other studies done by [8,10,19,21].

Irrespective of the dentition status, the pattern in the magnitude of BCPN and LCPN observed along the mandibular arch was similar although statistically not significant (*p* > 0.05) (Table 2). The LCPN at the 1st premolar region of the right side was an exception, with a statistically significant (*p* < 0.05) difference among all three groups, where edentulous had the minimum, partially edentulous showing intermediate and dentulous had the maximum value (Table 2). This clearly depicts the influence of dentition on the bone resorption. It has also been reported in the literature that the bone resorption which resulted from tooth loss, exceeded the buccal aspect in contrast to lingual [36,37].

With regard to the ACN dimension, astatistically significant difference (*p* < 0.001) was found in relation to all posterior teeth on comparing the study groups (Table 2). The natural phenomena of bone resorption in the absence of dentition can be a logical explanation for the pattern observed in the current study, where the edentulous subjects recorded the least and dentulous found with the maximum dimension among the group [36].

**Figure 7:**
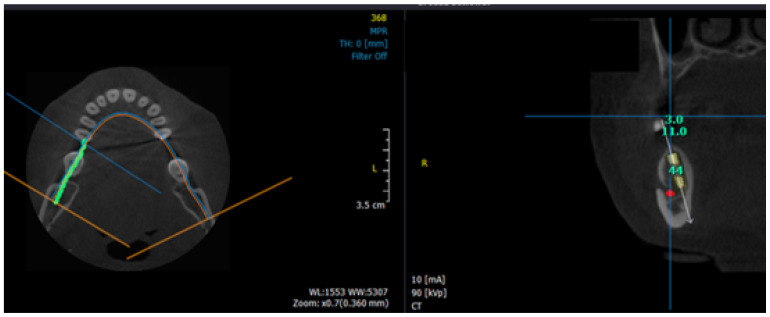
Inferior alveolar nerve tracing on axial plane view of CBCT and virtual implant placement by bypassing inferior alveolar nerve in cross-sectional view.

The BCPN and LCPN data of the present study has thrown light on phenomenal information. The mean value of LCPN in the first and second premolar region across the groups is more than 6 mm. This undoubtedly gives enough space for an implant placement towards the lingual cortical plate, thus circumventing the IAN and so the potential complication of nerve damage in implant placement cases was tried conventionally (Figure 7).

### 4.2. Comparison of BCPN, LCPN and ACN Dimensions among the Patients with Different Dentition Status and Side of the Jaw

Despite the human body being considered bilaterally symmetrical, there are variations seen with respect to various anatomical structures including visceral organs. With an intention to probe the variation between the sides of arches, all three dimensions were subjected to comparison. We found, that by large the resorption was greater on the left side of the jaw, specifically with respect to BCPN and ACN (*p* < 0.05 and *p* < 0.01) resulting in more available bone on the right side (Table 2). However, no variation was seen with respect to our areas of interest in LCPN dimension in the premolar region. Studies are available with observations supporting [10], as well as opposing [38], the results of the current study. The larger sample in the present study might be the reason for documenting variation in the side of arches.

### 4.3. Comparison of BCPN, LCPN and ACN Dimensions among the Patients with Different Dentition Status and Gender

The influence of gender on the three major parameters (BCPN, LCPN and ACN) of both sides of the jaw among the study groups was evaluated. This exhaustive analysis gave us about 90 paired comparisons, of which only 21 were statistically significant at varying (*p* < 0.05/0.01) confidence interval. The larger picture which emerged after the regress analysis was that the females were found to be more susceptible to bone resorption. Closer observation was then made in the LCPN dimension of the premolar region. Statistically significant variation was observed in the 2nd premolar region of edentulous patients (*p* < 0.05) and 1st premolar region of dentulous subjects (*p* < 0.05) (Table 3). In either of the locations, males scored higher in available bone in contrast to females. It is noteworthy to mention that despite the significant variation, both categories of gender have maintained a mean LCPN above 6 mm. This indicates that the proposed clinical approach of bypassing the IAN while placing implants in the lingual region of premolars still remains the preferred location (Figure 5). However, it is advisable to be a little cautious while performing this approach with female patients, because of the effect of fluctuating pattern of bone resorption under hormonal influence.

Conflicting results have been presented in the literature regarding the variation of BCPN and LCPN dimensions among genders. Whereas Shokry SM et al. have [8,19] found no significant difference among the genders, others [10] have found significant differences in the female but not in male in the dentate individuals.

### 4.4. Comparison of Buccal-Lingual-Coronal Dimensions among Patients with Different Dentition Status and Age Groups

Comparative study was made of the groups comprising of partially edentulous and dentulous patients with three subcategories in each group. The progressive decreasing and increasing trend in BCPN and LCPN, respectively, with the anterior advancement along the arch was observed unanimously in all age groups. This re-establishes the lingual course of the IAN in the molar region and buccal course in the premolars segment. Lower measurements of all three dimensions in the advanced age group was not a matter of surprise, as the periodontal status is usually found to be more compromised in that age group. As far as the younger and middle age groups are concerned a non-uniform pattern was reported. The cumulative analysis of these observations suggests that with the advancing age, the IAN will appear to be placed more lingually and superiorly in the molar region. Our results were found to be in agreement with previous researchers [10]. Careful analyses were made to scrutinize the possibility of lingual placement of dental implants in the premolar region of the both sides of the two study groups. Even with the statistically significant (*p* < 0.001) variation in this region, all age groups managed to have LCPN measurements greater than 6 mm (Table 4). This advocates the applicability of the proposed clinical approach of avoiding the IAN while placing implants in premolar regions in any age group aided with surgical guidance taking into consideration that angled abutment should be used during prosthesis fabrication. Nonetheless, the variation in the edentulous arches and systemic status of patients’ needs additional diagnostic workup.

There are a few limitations to the current study. The tracing of the IAN was done regardless of considering the reason for extraction in the partially edentulous subjects, as the amount of vertical bone loss might influence the course and position of the dental foramen. Nevertheless, a cutoff duration of two years for the edentulous area was considered to ensure the standardization of vertical bone height. This can be a potential aspect for future research.

In keeping with the observations of the present study, the null hypothesis was rejected, which states that there is an equal amount of bucco-lingual bone in the posterior region of the mandibular arch in all types of dentate states in patients to place a dental implant.

With the confinements of the present study, a clinical trial with larger sample size, preferable with prior sample size calculation, can give an insight in to the proposed outcome of taking a lingual approach in the mandibular premolar region irrespective of the dentition status.

## 5. Conclusions

The present study has come up with a conclusion that in the mandibular premolar lingual region, irrespective of the dentition status, a minimum of 6 mm bone is present to place the dental implant. However, the pre-operative evaluation of the surgical site with CBCT will remain a key prerequisite for implant placement. It was found that the IAN is more lingually placed in the molar region and moves toward the buccal side as it extendsinto the premolar region. There was minor variation seen in the gender, age and side in the various dentition statuses in respect to the dimensions of buccal, lingual and coronal bone available around the IAN. The data set used in the current studywill be made available on request from Dr. Mohammed G Sghaireen; dr.mohammed.sghaireen@jodent.org.

## Figures and Tables

**Table 1:** Descriptive analysis of biographic data of study groups expressed as *n* (%) except for the “age” variable.

Variable	Response	Study Group			Total
		Study Group I—Completely Edentulous (*n* = 80)	Study Group II—Partially Edentulous (*n* = 80)	Study Group III—Dentulous (*n* = 80)	
Age (Mean ± SD)		58.41 ± 2.928	41.54 ± 12.702	38.83 ± 14.389	-
Age	18–35 Years	-	28 (35)	35 (43.8)	63 (26.25)
	36–53 Years	-	28 (35)	24 (30)	52 (21.66)
	≥54 Years	80 (100)	24 (30)	21 (26.3)	125 (52.08)
Gender	Male	40 (50)	43 (53.8)	44 (55)	127 (52.91)
	Female	40 (50)	37 (46.3)	36 (45)	113 (47.08)

SD = standard deviation.

**Table 2:** BCPN, LCPN and ACN dimensions of right and left side of jaws measured in millimeters (mm) and presented as mean ±SD.

			Intragroup Comparison						Intergroup Comparison
Dimension	Tooth Position	Side of Jaw	Study Groups						*p* Value
			Study Group I	*p* Value	Study Group II	*p* Value	Study Group III	*p* Value	
BCPN	3rd Molar	Right	5.90 ± 1.16	0.056 ^b^	6.05 ± 1.04	0.005 **^b^	6.23 ± 1.14	0.130 ^b^	0.141 ^a^
		Left	5.50 ± 1.17		5.85 ± 0.90		6.19 ± 0.99		0.491 ^a^
	2nd Molar	Right	5.17 ± 1.261	0.230 ^b^	5.36 ± 1.04	0.015 *^b^	5.54 ± 1.09	0.884 ^b^	0.122 ^a^
		Left	5.12 ± 1.32		5.20 ± 0.80		5.52 ± 1.04		0.978 ^a^
	1st Molar	Right	3.70 ± 1.33	0.209 ^b^	3.70 ± 1.00	0.117 ^b^	3.87 ± 1.29	0.338 ^b^	0.594 ^a^
		Left	3.90 ± 1.19		3.95 ± 0.86		4.05 ± 1.07		0.654 ^a^
	2nd Premolar	Right	2.03 ± 1.13	0.042 *^b^	2.08 ± 0.56	0.899 ^b^	2.14 ± 0.65	0.002 **^b^	0.847 ^a^
		Left	1.48 ± 0.56		1.70 ± 0.72		1.74 ± 0.53		0.512 ^a^
	1st Premolar	Right	0	-	0	-	0	-	-
		Left	0		1.60 ± 0		0		-
LCPN	3rd Molar	Right	1.90 ± 0.65	0.853 ^b^	1.82 ± 0.76	0.312 ^b^	2.07 ± 0.65	0.795 ^b^	0.070 ^a^
		Left	1.92 ± 0.60		2.01 ± 1.45		2.04 ± 0.64		0.714 ^a^
	2nd Molar	Right	2.40 ± 0.72	0.286 ^b^	2.53 ± 0.93	0.533 ^b^	2.79 ± 0.85	0.616 ^b^	0.113 ^a^
		Left	2.42 ± 0.63		2.48 ± 0.71		2.73 ± 0.76		0.015 ^a^*
	1st Molar	Right	3.82 ± 1.14	0.583 ^b^	4.00 ± 1.24	0.475 ^b^	4.27 ± 1.28	0.238 ^b^	0.070 ^a^
		Left	3.73 ± 1.07		3.87 ± 1.02		4.05 ± 1.08		0.165 ^a^
	2nd Premolar	Right	6.32 ± 1.90	0.987 ^b^	6.51 ± 1.62	0.095 ^b^	6.68 ± 1.75	0.906 ^b^	0.203 ^a^
		Left	6.09 ± 1.67		6.35 ± 1.44		6.52 ± 1.55		0.080 ^a^
	1st Premolar	Right	6.50 ± 1.20	0.298 ^b^	6.80 ± 1.46	0.364 ^b^	7.61 ± 1.34	0.302 ^b^	0.044 ^a^*
		Left	6.15 ± 1.17		6.44 ± 1.09		7.53 ± 0.77		0.286 ^a^
ACN	3rd Molar	Right	14.41 ± 1.39	0.500 ^b^	16.30 ± 2.07	0.011 ^b^*	16.68 ± 2.16	0.945 ^b^	0.000 ^a^***
		Left	16.90 ± 2.09		16.11 ± 2.42		14.39 ± 1.95		0.000 ^a^***
	2nd Molar	Right	14.98 ± 1.75	0.954 ^b^	17.98 ± 2.31	0.007 ^b^**	17.80 ± 2.36	0.952 ^b^	0.000 ^a^***
		Left	17.78 ± 2.12		16.96 ± 2.43		14.96 ± 2.39		0.000 ^a^***
	1st Molar	Right	15.59 ± 2.45	0.727 ^b^	18.54 ± 2.65	0.003 ^b^**	18.95 ± 2.48	0.749 ^b^	0.000 ^a^***
		Left	18.82 ± 2.28		17.01 ± 3.75		15.73 ± 2.76		0.000 ^a^***
	2nd Premolar	Right	12.49 ± 1.34	0.864 ^b^	13.34 ± 0.75	0.008 ^b^**	13.80 ± 1.64	0.686 ^b^	0.000 ^a^***
		Left	13.84 ± 1.46		13.00 ± 0.82		12.41 ± 1.32		0.000 ^a^***
	1st Premolar	Right	8.85 ± 1.42	0.989 ^b^	10.66 ± 0.91	0.563 ^b^	11.88 ± 2	0.763 ^b^	0.000 ^a^***
		Left	11.87 ± 1.49		10.46 ± 0.89		8.96 ± 1.08		0.000 ^a^***

SD = standard deviation; BCPN = buccal cortical plate to nerve dimension; LCPN = lingual cortical plate to nerve dimension; ACN = alveolar crest to nerve dimension. Note: * *p* value < 0.05; ** *p* value < 0.01; *** *p* value < 0.001; a = one way ANOVA; b = independent *t*-test. The figures in bold means that they are statistically significant.

**Table 3:** BCPN, LCPN and ACN dimensions compared among gender measured in millimeters (mm) and presented as mean ±SD.

Side of Arch	Dimension	Tooth #	Study Group I			Study Group II			Study Group III		
			Male	Female	*p* Value	Male	Female	*p* Value	Male	Female	*p* Value
Right	BCPN	3rd Molar	6.02 ± 1.04	5.85 ± 1.31	0.520	6.20 ± 1.10	5.57 ± 0.85	0.006 **	6.07 ± 0.99	6.38 ± 1.27	0.231
		2nd Molar	5.36 ± 1.23	5.16 ± 1.29	0.475	5.43 ± 1.18	4.90 ± 0.78	0.024 *	5.48 ± 0.94	5.60 ± 1.24	0.648
		1st Molar	3.82 ± 1.40	3.55 ± 1.22	0.372	3.65 ± 1.17	3.75 ± 0.78	0.678	3.96 ± 1.30	3.78 ± 1.29	0.535
		2nd Premolar	2.25 ± 1.34	1.88 ± 0.83	0.367	2.10 ± 0.55	1.98 ± 0.58	0.548	2.27 ± 0.43	2.00 ± 0.82	0.170
		1st Premolar	0	0	-	0	0	-	0	0	-
	LCPN	3rd Molar	1.85 ± 0.65	1.97 ± 0.66	0.415	1.51 ± 0.67	2.18 ± 0.69	0.000 ***	2.17 ± 0.73	1.97 ± 0.55	0.169
		2nd Molar	2.57 ± 0.79	2.49 ± 0.64	0.652	2.07 ± 0.77	2.79 ± 0.96	0.000 ***	3.11 ± 0.96	2.47 ± 0.59	0.001 **
		1st Molar	4.05 ± 1.36	3.55 ± 0.72	0.053	3.89 ± 1.32	4.12 ± 1.13	0.427	4.37 ± 1.43	4.17 ± 1.13	0.476
		2nd Premolar	6.92 ± 2.13	6.03 ± 1.46	0.036 *	6.62 ± 0.71	6.36 ± 1.50	0.500	6.07 ± 1.88	6.09 ± 1.62	0.970
		1st Premolar	6.21 ± 0.04	6.94 ± 1.43	0.235	6.82 ± 1.62	6.77 ± 1.29	0.944	7.83 ± 1.49	6.71 ± 0.73	0.012 *
	ACN	3rd Molar	14.94 ± 1.21	13.89 ± 1.38	0.001 **	17.22 ± 2.13	16.81 ± 1.99	0.386	16.83 ± 2.19	16.49 ± 2.13	0.489
		2nd Molar	15.54 ± 1.60	14.42 ± 1.73	0.004 **	18.24 ± 2.38	17.68 ± 2.23	0.281	18.20 ± 2.39	17.31 ± 2.26	0.094
		1st Molar	15.79 ± 2.55	15.40 ± 2.36	0.475	19.34 ± 2.01	17.62 ± 3.02	0.003 **	19.75 ± 2.30	17.97 ± 2.35	0.001 **
		2nd Premolar	12.57 ± 1.37	12.42 ± 1.32	0.626	13.45 ± 0.75	13.21 ± 0.74	0.181	13.81 ± 1.23	13.78 ± 2.04	0.931
		1st Premolar	7.39 ± 0.06	9.63 ± 1.14	0.000 ***	10.07 ± 0.58	11.50 ± 0.54	0.002 **	11.14 ± 0.70	12.25 ± 2.11	0.234
Left	BCPN	3rd Molar	6.27 ± 1.10	6.33 ± 1.27	0.837	6.44 ± 0.96	6.24 ± 0.83	0.310	6.37 ± 1.11	6.60 ± 0.87	0.317
		2nd Molar	5.57 ± 1.21	5.45 ± 1.46	0.673	5.66 ± 0.78	5.42 ± 0.82	0.186	5.53 ± 1.24	5.50 ± 0.82	0.890
		1st Molar	3.98 ± 1.11	3.93 ± 1.30	0.0848	4.02 ± 0.89	3.76 ± 0.81	0.168	3.97 ± 1.11	4.13 ± 1.04	0.497
		2nd Premolar	0.99 ± 0.06	1.85 ± 0.47	0.000 ***	2.08 ± 0.81	1.92 ± 0.58	0.471	1.72 ± 0.59	1.76 ± 0.50	0.804
		1st Premolar	0	0	-	1.60 ± 0.0	0	-	0	0	-
	LCPN	3rd Molar	1.87 ± 0.60	1.98 ± 0.59	0.398	1.73 ± 0.58	2.32 ± 2.01	0.070	2.01 ± 0.62	2.07 ± 0.66	0.668
		2nd Molar	2.32 ± 0.60	2.54 ± 0.66	0.132	2.36 ± 0.64	2.63 ± 0.76	0.089	2.69 ± 0.88	2.77 ± 0.62	0.640
		1st Molar	3.90 ± 1.11	3.52 ± 0.98	0.114	3.73 ± 0.96	4.03 ± 1.07	0.183	3.93 ± 0.91	4.17 ± 1.22	0.325
		2nd Premolar	6.76 ± 2.11	6.22 ± 0.79	0.158	6.12 ± 1.65	6.07 ± 1.17	0.878	6.58 ± 1.14	6.25 ± 1.17	0.828
		1st Premolar	6.10 ± 0.14	6.16 ± 1.27	0.950	6.24 ± 0.73	6.78 ± 1.52	0.257	7.88 ± 0.84	7.43 ± 0.65	0.109
	ACN	3rd Molar	15.03 ± 1.95	13.76 ± 1.77	0.003 **	16.26 ± 2.47	15.94 ± 2.37	0.564	17.47 ± 2.13	16.21 ± 1.84	0.007 **
		2nd Molar	15.51 ± 2.30	14.41 ± 2.37	0.039 *	17.03 ± 2.52	16.86 ± 2.36	0.758	18.37 ± 1.97	17.04 ± 2.10	0.005 **
		1st Molar	16.05 ± 2.89	15.41 ± 2.61	0.302	17.93 ± 2.69	15.94 ± 4.50	0.017 *	19.33 ± 1.98	18.19 ± 2.50	0.027 *
		2nd Premolar	12.41 ± 1.41	12.40 ± 1.25	0.967	13.08 ± 0.83	12.92 ± 0.80	0.377	13.72 ± 1.18	13.99 ± 1.76	0.417
		1st Premolar	8.85 ± 1.01	9.03 ± 1.15	0.710	10.04 ± 0.80	11.30 ± 0.12	0.004 **	11.50 ± 0.70	11.93 ± 1.59	0.723

SD = standard deviation; BCPN = buccal cortical plate to nerve dimension; LCPN = lingual cortical plate to nerve dimension; ACN = alveolar crest to nerve dimension. Note: * *p* value < 0.05; ** *p* value < 0.01; *** *p* value < 0.001. The figures in bold means that they are statistically significant.

**Table 4:** BCPN, LCPN and ACN dimensions compared betweenthe age categories in study groups measured in millimeters (mm) and presented asmean ±SD.

Side of Arch	Dimension	Tooth #	Study Group II				Study Group III			
			Age Categories			*p* Value	Age Categories			*p* Value
			18–35 Years	36–53 Years	≥ 54 Years		18–35 Years	36–53 Years	≥ 54 Years	
Right	BCPN	3rd Molar	5.99 ± 1.42	5.91 ± 0.89	5.83 ± 0.80	0.859	6.70 ± 1.03	6.12 ± 0.83	5.56 ± 1.33	0.028 *
		2nd Molar	5.30 ± 1.39	5.12 ± 0.92	5.16 ± 0.82	0.808	5.62 ± 1.14	5.57 ± 1.13	4.86 ± 1.31	0.032 *
		1st Molar	3.66 ± 1.26	3.44 ± 1.04	3.59 ± 0.70	0.633	3.82 ± 1.28	3.98 ± 1.48	3.44 ± 1.22	0.283
		2nd Premolar	2.17 ± 0.67	2.08 ± 0.45	1.67 ± 0.13	0.144	2.37 ± 1.55	2.14 ± 0.96	1.76 ± 0.67	0.437
		1st Premolar	-	-	-		-	-	-	
	LCPN	3rd Molar	2.13 ± 0.84	1.81 ± 0.66	1.56 ± 0.69	0.023 *	2.17 ± 0.74	2.10 ± 0.70	1.87 ± 0.52	0.090
		2nd Molar	2.66 ± 0.87	2.44 ± 0.97	2.14 ± 0.90	0.134	2.50 ± 0.84	2.57 ± 0.59	2.43 ± 0.65	0.789
		1st Molar	4.20 ± 1.33	4.15 ± 1.35	3.96 ± 0.97	0.09	3.86 ± 0.84	4.23 ± 1.12	3.53 ± 1.24	0.063
		2nd Premolar	6.56 ± 1.80	6.35 ± 1.64	6.16 ± 1.80	0.191	6.60 ± 1.49	6.15 ± 1.49	6.24 ± 1.78	0.004 **
		1st Premolar	6.83 ± 0.38	6.22 ± 0.24	6.03 ± 0.38	0.04 *	6.51 ± 0.15	6.32 ± 1.61	6.06 ± 0.64	0.025 *
	ACN	3rd Molar	17.52 ± 1.41	18.21 ± 1.27	15.09 ± 2.13	0.000 ***	16.72 ± 1.33	17.44 ± 2.39	15.73 ± 2.82	0.027 *
		2nd Molar	17.51 ± 1.57	19.27 ± 1.56	16.86 ± 2.37	0.007 **	17.65 ± 1.71	18.68 ± 2.34	17.04 ± 3.04	0.059
		1st Molar	18.14 ± 3.15	19.12 ± 1.64	17.17 ± 2.04	0.000 ***	18.38 ± 1.83	19.34 ± 2.46	18.30 ± 2.86	0.031 *
		2nd Premolar	13.58 ± 0.51	13.25 ± 1.02	13.16 ± 0.60	0.101	13.93 ± 2.01	13.75 ± 1.61	13.61 ± 0.84	0.773
		1st Premolar	10.90 ± 0.00	10.88 ± 1.11	9.55 ± 0.00	0.169	12.88 ± 2.94	11.25 ± 0,82	11.50 ± 0.30	0.259
Left	BCPN	3rd Molar	6.28 ± 1.20	6.16 ± 0.85	6.20 ± 0.61	0.310	6.60 ± 1.24	6.38 ± 0.76	6.00 ± 1.31	0.093
		2nd Molar	5.46 ± 0.69	5.88 ± 0.85	5.27 ± 0.77	0.180	5.88 ± 1.31	5.40 ± 0.94	5.28 ± 1.49	0.091
		1st Molar	3.94 ± 0.95	3.58 ± 0.57	3.28 ± 0.57	0.028 *	3.47 ± 1.27	3.80 ± 0.99	3.25 ± 1.21	0.067
		2nd Premolar	1.95 ± 0.50	2.06 ± 0.97	1.85 ± 0.65	0.175	1.91 ± 0.67	1.66 ± 0.22	1.31 ± 0.67	0.042 *
		1st Premolar	-	-	-	-	-	-	-	
	LCPN	3rd Molar	2.30 ± 0.79	2.15 ± 0.71	2.11 ± 2.21	0.243	1.82 ± 0.54	1.66 ± 0.64	1.42 ± 0.54	0.010 *
		2nd Molar	2.87 ± 0.84	2.38 ± 0.72	2.25 ± 0.38	0.004 **	2.93 ± 0.57	2.27 ± 0.57	2.02 ± 0.57	0.002 **
		1st Molar	4.01 ± 1.05	3.85 ± 1.14	3.76 ± 0.88	0.681	3.29 ± 0.70	4.85 ± 1.09	3.18 ± 0.37	0.000 ***
		2nd Premolar	6.25 ± 0.90	6.15 ± 1.59	6.05 ± 1.76	0.774	6.53 ± 0.68	6.24 ± 1.54	6.08 ± 1.47	0.01 *
		1st Premolar	6.58 ± 0.00	6.22 ± 1.64	6.37 ± 0.81	0.176	6.40 ± 0.82	6.11 ± 0.96	6.03 ± 0.82	0.04 *
	ACN	3rd Molar	16.69 ± 1.80	17.15 ± 2.36	15.22 ± 2.08	0.009 **	16.76 ± 1.15	17.56 ± 2.37	16.40 ± 2.79	0.152
		2nd Molar	17.66 ± 1.74	18.00 ± 2.02	14.92 ± 2.40	0.000 ***	17.47 ± 2.46	17.52 ± 2.43	17.45 ± 1.54	0.126
		1st Molar	16.36 ± 1.72	16.79 ± 5.38	15.36 ± 1.72	0.366	18.59 ± 2.21	19.60 ± 0.98	18.41 ± 1.61	0.128
		2nd Premolar	12.82 ± 0.69	13.53 ± 0.56	12.82 ± 0.69	0.04 *	14.07 ± 1.72	13.63 ± 1.47	13.69 ± 0.88	0.473
		1st Premolar	10.30 ± 1.22	10.22 ± 0.00	9.08 ± 1.22	0.669	12.54 ± 1.65	11.91 ± 0.60	10.23 ± 0.00	0.065

SD = standard deviation; BCPN = buccal cortical plate to nerve dimension; LCPN =lingual cortical plate to nerve dimension; ACN = alveolar crest to nerve dimension. Note: * *p* value < 0.05; ** *p* value < 0.01; *** *p* value < 0.001. The figures in bold, means that they are statistically significant.

## References

[1] Weiss R., Read-Fuller A.M. (2019). Cone Beam Computed Tomography in Oral and Maxillofacial Surgery: An Evidence-Based Review. Dent. J..

[2] Benavides E., Rios H.F., Ganz S.D., An C.-H., Resnik R., Reardon G.T., Feldman S.J., Mah J.K., Hatcher D., Kim M.-J. (2012). Use of Cone Beam Computed Tomography in Implant Dentistry. Implant. Dent..

[3] Srivastava K.C., Ijar, Shrivastava D., Austin D. (2016). Journey towards the 3d dental imaging- the milestones in the advancement of dental imaging. Int. J. Adv. Res..

[4] Jacobs R., Salmon B., Codari M., Hassan B., Bornstein M.M. (2018). Cone beam computed tomography in implant dentistry: Recommendations for clinical use. BMC Oral Heal..

[5] Massey N.D., A Galil K., Wilson T.D. (2013). Determining position of the inferior alveolar nerve via anatomical dissection and micro-computed tomography in preparation for dental implants. J. Can. Dent. Assoc..

[6] Murat S., Kamburoglu K., Kilic C., Özen T., Gurbuz A. (2014). Nerve Damage Assessment Following Implant Placement in Human Cadaver Jaws: An Ex Vivo Comparative Study. J. Oral Implant..

[7] Yatzkair G., Cheng A., Brodie S., Raviv E., Boyan B., Schwartz Z. (2014). Accuracy of computer-guided implantation in a human cadaver model. Clin. Oral Implant. Res..

[8] Balaji S.M., Krishnaswamy N.R., Kumar S.M., Rooban T. (2012). Inferior alveolar nerve canal position among South Indians: A cone beam computed tomographic pilot study. Ann. Maxillofac. Surg..

[9] Khorshidi H., Raoofi S., Ghapanchi J., Shahidi S., Paknahad M. (2016). Cone Beam Computed Tomographic Analysis of the Course and Position of Mandibular Canal. J. Maxillofac. Oral Surg..

[10] Shokry S.M., Alshaib S.A., Al Mohaimeed Z.Z., Ghanimah F., Altyebe M.M., Alenezi M.A., Shadd F., Aldali S.Z., Alotaibi M.M. (2018). Assessment of the Inferior Alveolar Nerve Canal Course Among Saudis by Cone Beam Computed Tomography (Pilot Study). J. Maxillofac. Oral Surg..

[11] Aljarbou F.A., Aldosimani M.A., Althumairy R.I., Alhezam A.A., Aldawsari A.I. (2019). An analysis of the first and second mandibular molar roots proximity to the inferior alveolar canal and cortical plates using cone beam computed tomography among the Saudi population. Saudi Med. J..

[12] Cartes G., Garay I., Deana N.F., Navarro P., Alves N. (2018). Mandibular Canal Course and the Position of the Mental Foramen by Panoramic X-Ray in Chilean Individuals. BioMed Res. Int..

[13] Al-Mahalawy H.A., Al-Aithan H., Al-Kari B., Al-Jandan B., Shujaat S. (2017). Determination of the position of mental foramen and frequency of anterior loop in Saudi population. A retrospective CBCT study. Saudi Dent. J..

[14] Alam M.K., Alhabib S., AlZarea B., Irshad M., Faruqi S., Sghaireen M.G., Patil S.R., Basri R. (2017). 3D CBCT morphometric assessment of mental foramen in Arabic population and global comparison: Imperative for invasive and non-invasive procedures in mandible. Acta Odontol. Scand..

[15] Demir A., Izgi E., Pekiner F. (2015). Anterior Loop of the Mental foramen in a Turkish Subpopulation with Dentate Patients: A Cone Beam Computed Tomography Study. J. Marmara Univ. Inst. Heal. Sci..

[16] Greenstein G., Tarnow D. (2006). The Mental Foramen and Nerve: Clinical and Anatomical Factors Related to Dental Implant Placement: A Literature Review. J. Periodontol..

[17] Wei X., Gu P., Hao Y., Wang J. (2019). Detection and characterization of anterior loop, accessory mental foramen, and lateral lingual foramen by using cone beam computed tomography. J. Prosthet. Dent..

[18] Lin M.-H., Mau L.-P., Cochran D.L., Shieh Y.-S., Huang P.-H., Huang R.-Y. (2014). Risk assessment of inferior alveolar nerve injury for immediate implant placement in the posterior mandible: A virtual implant placement study. J. Dent..

[19] Mirbeigi S., Safaee A., Ezoddini F., Khojastepour L., Navab-Azam A. (2016). Buccolingual course of the inferior alveolar canal in different mental foramen locations: A cone beam computed tomography study of an Iranian population. Int. J. Appl. Basic Med Res..

[20] Velasco-Torres M., Padial-Molina M., Avila-Ortiz G., García-Delgado R., Catena A., Galindo-Moreno P. (2017). Inferior alveolar nerve trajectory, mental foramen location and incidence of mental nerve anterior loop. Medicina Oral Patología Oral y Cirugia Bucal.

[21] Kavarthapu A., Thamaraiselvan M. (2018). Assessing the variation in course and position of inferior alveolar nerve among south Indian population: A cone beam computed tomographic study. Indian J. Dent. Res..

[22] Juodzbalys G., Wang H.-L. (2010). Identification of the Mandibular Vital Structures: Practical Clinical Applications of Anatomy and Radiological Examination Methods. J. Oral Maxillofac. Res..

[23] Von Elm E., Altman D.G., Egger M., Pocock S.J., Gøtzsche P.C., Vandenbroucke J.P. (2007). The Strengthening the Reporting of Observational Studies in Epidemiology (STROBE) Statement. Epidemiology.

[24] Al-Omiri M., Sghaireen M.G., Alhijawi M.M., Alzoubi I.A., Lynch C.D., Lynch E. (2014). Maximum bite force following unilateral implant-supported prosthetic treatment: Within-subject comparison to opposite dentate side. J. Oral Rehabil..

[25] Na J.Y., Han S.-S., Jeon K.J., Choi Y.J., Choi S.-H., Lee C. (2019). Prognosis in case of nerve disturbance after mandibular implant surgery in relation to computed tomography findings and symptoms. J. Periodontal Implant. Sci..

[26] Liang X., Jacobs R., Hassan B., Li L., Pauwels R., Corpas L., Souza P.H.C., Martens W., Shahbazian M., Alonso A. (2010). A comparative evaluation of Cone Beam Computed Tomography (CBCT) and Multi-Slice CT (MSCT). Eur. J. Radiol..

[27] Monsour P., Dudhia R. (2008). Implant radiography and radiology. Aust. Dent. J..

[28] Agbaje J., Van De Casteele E., Salem A.S., Anumendem D., Lambrichts I., Politis C. (2016). Tracking of the inferior alveolar nerve: Its implication in surgical planning. Clin. Oral Investig..

[29] Angel J.S., Mincer H.H., Chaudhry J., Scarbecz M. (2011). Cone-beam Computed Tomography for Analyzing Variations in Inferior Alveolar Canal Location in Adults in Relation to Age and Sex*. J. Forensic Sci..

[30] Ali S.P., Gupta J. (2013). Cone beam computed tomography in oral implants. Natl. J. Maxillofac. Surg..

[31] Worthington P. (2004). Injury to the inferior alveolar nerve during implant placement: A formula for protection of the patient and clinician. Int. J. Oral Maxillofac. Implant..

[32] Juodzbalys G., Wang H.-L., Sabalys G. (2011). Injury of the Inferior Alveolar Nerve during Implant Placement: A Literature Review. J. Oral Maxillofac. Res..

[33] Gurler G., Tufekcioglu S., Delilbasi C., Dilaver E., Ozer N. (2017). Is 2 mm a safe distance from the inferior alveolar canal to avoid neurosensory complications in implant surgery?. Niger. J. Clin. Pr..

[34] Sammartino G., Marenzi G., Citarella R., Ciccarelli R., Wang H.-L. (2008). Analysis of the Occlusal Stress Transmitted to the Inferior Alveolar Nerve by an Osseointegrated Threaded Fixture. J. Periodontol..

[35] Froum S.J., Casanova L., Byrne S., Cho S.-C. (2011). Risk Assessment before Extraction for Immediate Implant Placement in the Posterior Mandible: A Computerized Tomographic Scan Study. J. Periodontol..

[36] Cho H.-J., Jeon J.-Y., Ahn S.-J., Lee S.-W., Chung J.-R., Park C.-J., Hwang K.-G. (2019). The preliminary study for three-dimensional alveolar bone morphologic characteristics for alveolar bone restoration. Maxillofac. Plast. Reconstr. Surg..

[37] Hansson S., Halldin A. (2012). Alveolar ridge resorption after tooth extraction: A consequence of a fundamental principle of bone physiology. J. Dent. Biomech..

[38] Wong S.K., Patil P.G. (2018). Measuring anterior loop length of the inferior alveolar nerve to estimate safe zone in implant planning: A CBCT study in a Malaysian population. J. Prosthet. Dent..

